# Corrosion Resistance of Coatings Based on Chromium and Aluminum of Titanium Alloy Ti-6Al-4V

**DOI:** 10.3390/ma17153880

**Published:** 2024-08-05

**Authors:** Tetiana Loskutova, Michael Scheffler, Ivan Pavlenko, Kamil Zidek, Inna Pohrebova, Nadiia Kharchenko, Iryna Smokovych, Oleksandr Dudka, Volodymyr Palyukh, Vitalii Ivanov, Yaroslav Kononenko

**Affiliations:** 1 Department of Physical Materials Science and Heat Treatment, Y.O. Paton Institute of Materials Science and Welding, National Technical University of Ukraine “Igor Sikorsky Kyiv Polytechnic Institute”, 37 Beresteiskyi Ave., 03056 Kyiv, Ukraine; losktv@ukr.net (T.L.); adudka@bigmir.net (O.D.); konoyaroslav@gmail.com (Y.K.); 2 Faculty of Mechanical Engineering, Institute for Materials and Joining Technology, Otto-von-Guericke University Magdeburg, 2 Universitätsplatz, 39106 Magdeburg, Germany; m.scheffler@ovgu.de; 3 Department of Computational Mechanics Named after Volodymyr Martsynkovskyy, Faculty of Technical Systems and Energy Efficient Technologies, Sumy State University, 116 Kharkivska St., 40007 Sumy, Ukraine; i.pavlenko@cm.sumdu.edu.ua; 4 Department of Industrial Engineering and Informatics, Faculty of Manufacturing Technologies with a Seat in Presov, Technical University of Kosice, 1 Bayerova St., 08001 Presov, Slovakia; 5 Department of Electrochemical Production Technology, Faculty of Chemical Technology, National Technical University of Ukraine “Igor Sikorsky Kyiv Polytechnic Institute”, 37 Beresteiskyi Ave., 03056 Kyiv, Ukraine; i.pogrebova@kpi.ua; 6 Department of Applied Materials Science and Technology of Constructional Materials, Faculty of Technical Systems and Energy Efficient Technologies, Sumy State University, 116 Kharkivska St., 40007 Sumy, Ukraine; n.harchenko@pmtkm.sumdu.edu.ua; 7 thyssenkrupp Materials Trading GmbH, 1 Thyssenkrupp Allee, 46143 Essen, Germany; iryna.smokovych@thyssenkrupp-materials.com; 8 Department of Strength of Materials and Structural Mechanics, Institute of Civil Engineering and Building Systems, Lviv Polytechnic National University, 2 Karpinskoho St., 79013 Lviv, Ukraine; volodymyr.m.palyukh@gmail.com; 9 Department of Manufacturing Engineering, Machines and Tools, Faculty of Technical Systems and Energy Efficient Technologies, Sumy State University, 116 Kharkivska St., 40007 Sumy, Ukraine; ivanov@tmvi.sumdu.edu.ua; 10 Department of Automobile and Manufacturing Technologies, Faculty of Manufacturing Technologies with a Seat in Presov, Technical University of Kosice, 1 Bayerova St., 08001 Presov, Slovakia

**Keywords:** corrosion-resistant coating, functional layer, process innovation, chemical-thermal treatment, chromium, aluminum

## Abstract

Improvement of wear, corrosion, and heat-resistant properties of coatings to expand the operational capabilities of metals and alloys is an urgent problem for modern enterprises. Diffusion titanium, chromium, and aluminum-based coatings are widely used to solve this challenge. The article aims to obtain the corrosion-electrochemical properties and increase the microhardness of the obtained coatings compared with the initial Ti-6Al-4V alloy. For this purpose, corrosion resistance, massometric tests, and microstructural analysis were applied, considering various aggressive environments (acids, sodium carbonate, and hydrogen peroxide) at different concentrations, treatment temperatures, and saturation times. As a result, corrosion rates, polarization curves, and X-ray microstructures of the uncoated and coated Ti-6Al-4V titanium alloy samples were obtained. Histograms of corrosion inhibition ratio for the chromium–aluminum coatings in various environments were discussed. Overall, the microhardness of the obtained coatings was increased 2.3 times compared with the initial Ti-6Al-4V alloy. The corrosion-resistant chromaluminizing alloy in aqueous solutions of organic acids and hydrogen peroxide was recommended for practical application in conditions of exposure to titanium products.

## 1. Introduction

In the modern chemical industry, metal products are exposed to various aggressive environments: solutions of acids, alkalis, salts of multiple concentrations, organic compounds, etc. The simultaneous action of such environments and specific loads leads to significant corrosion of equipment and its premature failure [1,2,3].

To extend the service life and improve the performance of structures and devices, an important task is to find ways to modify the surface of metal products and parts [4,5].

Researchers actively carry out studies on up-to-date wear-resistant, corrosion-resistant, and heat-resistant coatings that can expand the operational capabilities of metals [6,7,8,9,10], alloys [11], and composites [12]. There is a wide range of methods and techniques for applying protection coatings, which have their characteristic features to ensure the formation of functional layers on the surface of metals and alloys that differ in composition, structure, density, and adhesion strength to the substrate. Among them, diffusion coatings based on nitrides, carbides, and intermetallic compounds of metals, i.e., containing titanium [13], aluminum [14] and chromium [15], and others [16,17], are widely used.

The application of diffusion coatings containing chromium and aluminum on the surface of carbon steels increases their heat resistance and corrosion resistance in various oxidizing environments [18]. The high thermodynamic activity of these elements and the formation of protective oxide films, such as Cr_2_O_3_ and Al_2_O_3_, on the surface of steels [19,20] significantly enhance the corrosion resistance and improve the operational properties of steel products.

Titanium and its alloys are widely used in the aerospace, automotive, and chemical industries [21,22]. This is due to their low density, high specific strength, and excellent corrosion resistance in most aggressive environments. However, they are characterized by low heat resistance (service temperatures do not exceed 400–500 °C), low wear resistance, and a tendency for stick and burr formation [23,24].

A wide range of robots is dedicated to surface modification of titanium and titanium-based alloys. Paper [25] provides an overview of existing alitization methods. Aluminum coatings increase corrosion resistance to oxidation due to the formation of a protective aluminum scale on the surface [26,27]. It is noteworthy that aluminum coatings are usually applied to surfaces by one of the following methods [28,29,30]: halide-activated pack cementation (HAPC), gas phase aluminization (GPA), suspension, and chemical vapor deposition (CVD).

In [31], alitization of Ti-6Al-4V alloy was carried out by the HAPC method in a mixture of powder in a chlorine environment: 10% wt. aluminum, 87% wt. Al2O3, and 3.0% wt. NH_4_Cl. The process was carried out in a vacuum at 1050 °C for 3 h. The resulting coating consists of layers based on Al_3_Ti, AlTi, and AlTi_3_. However, Ti-Al binary alloys with a significant aluminum content become extremely brittle, which is in good agreement with the data of [32]. The Al_3_Ti phase is characterized by high brittleness. The tetragonality of its lattice can explain this. This structure can easily be destroyed during operation.

It is possible to reduce the brittleness of the Al_3_Ti phase by introducing chromium into the coating. In [33], alitized coatings were compared. The coatings were applied to α titanium alloy TIMETAL 1100 and γ-TiAl-based Ti-48Al-2Cr-2Nb. As a result of the treatment, a diffusion coating of the same Al_3_Ti phase composition was formed on the surface of the alloys. However, on the alloy without chromium, the diffusion layer of Al_3_Ti was characterized by numerous cracks and chips, while the alloyed coating with chromium was characterized by continuity and ductility. Some researchers suggest that adding chromium can explain the decrease in coating brittleness. As a result, the phase composition of the AlTi_3_ coating (DO22 structure) changes to Ti(Al, Cr)_3_ (L12 structure) [34].

Ti-Al-Cr alloys with the participation of the Lavès phases Ti(Al, Cr)_2_ and σ-phase (Ti_0.25_Al_0.67_Cr_0.08_) are characterized by high heat resistance and mechanical properties [14,19]. First, it is an alloy containing 70–75% of the σ-phase and 25–30% of the Laves phase.

Despite this, diffusion saturation of titanium and titanium alloys with chromium or with chromium and aluminum has not yet been studied much [15,35,36,37]. In [15], it was determined that the microhardness of the surface of titanium alloys increases by 2–2.5 times after saturation with aluminum and chromium. The authors conclude that chromaluminizing improves the erosion and abrasion resistance of alloys. In addition, chromaluminizing is recommended for Ti-6Al-4V titanium alloy products intended for operation at temperatures of 500, 700, and 900 °C for 36 h [15].

The authors of [15] conclude that the simultaneous saturation of titanium alloy with chromium and aluminum is recommended to increase wear and heat resistance. However, to date, there are no studies on the effect of this type of coating on the corrosion resistance of titanium alloys.

Therefore, the development of methods for obtaining multilayer diffusion coatings containing chromium and aluminum (chromaluminizing) on the surface of the titanium alloy Ti-6Al-4V, the determination of their composition and structure, the investigation of their corrosion-electrochemical properties is of the current scientific and practical interest.

Therefore, the study’s primary purpose is to investigate the corrosion-electrochemical behavior of the original and chromaluminizing Ti-6Al-4V alloy.

## 2. Materials and Methods

The batch cementation of the Ti-6Al-4V alloy (89.05% Ti, 6.18% Al, 4.79% V) was carried out at a temperature of 1050 °C for 4 h in a saturating mixture of the following composition: Cr (28% wt.), Al (42% wt.), Al_2_O_3_ (25% wt.), and activator NH_4_Cl (5% wt.).

The chemical composition of the obtained coatings was examined by X-ray microspectral analysis using a CamScan 4D scanning electron microscope (Applied Beams LLC, Beaverton, OR, USA) and INCA-200 Energy microanalyzer (Blue Star Ltd., Mumbai, India). X-ray phase analysis was conducted on a Ultima-IV («Rigaku Corporation» Tokyo, Japan) using monochromatic Cu_Kα_ radiation. Microhardness and coating thickness is determined according to the standard ASTM E92-82(2003) [38] by pressing on the device LHVS-1000Z (Chongqing Leeb Instrument Co., Ltd., Chongqing, China). Indenter is a quadrangular diamond pyramid with an angle at the top of 136°. The characteristics of microhardness is determined as follows, kgf/mm^2^ [31]:

(1) $$H=1.854\frac{P}{D^{2}}$$

where *P* = load, g and *D*—imprint diagonal, μm.

Measurements of the thickness and microhardness of the diffusion layers were performed not less than in 15–20 view areas. The measurement error is 0.1 GPa

In this case, mass is determined by electronic scales “AXIS ANG220C” (“Himstatus” Ltd., Kharkiv, Ukraine). The error of massometric tests is 3–7%.

Corrosion studies of the initial samples and samples with coatings were performed using the massometric method at a temperature of 20 °C in air. The investigations were conducted in industrially relevant aggressive environments: 15% aqueous solution of acetic acid (CH_3_COOH), 1.5% aqueous solution of adipic acid (C_6_H_10_O_4_), 10% aqueous solution of oxalic acid (C_2_H_2_O_4_), 10% aqueous solution of sulfuric acid (H_2_SO_4_), 50% aqueous solution of phosphoric acid (H_3_PO_4_), 10% aqueous solution of sodium carbonate (Na_2_CO_3_), 40% aqueous solution of nitric acid (HNO_3_), and 3, 12, and 35% aqueous hydrogen peroxide solutions (H_2_O_2_). A relatively wide range of areas of application of the aggressive environments proposed in this paper is known. For example, acetic acid is used mainly in industrial organic synthesis. In addition, acetic acid and its esters are important industrial solvents and extractants. Adipic acid is one of the most important products of the chemical industry. It is a semi-product for the industrial production of synthetic fiber Nylon 66. The acid is also used in producing polyhexamethylene adipamide, its esters, polyurethanes, lubricants, plasticizers, and as a food additive. Nitric acid is one of the most important products of the chemical industry. It is produced in large quantities and used in the production of nitrogen fertilizers, in non-ferrous metallurgy for the separation of metals, and in the chemical industry for the production of plastics, explosives, celluloid and photographic film, artificial fiber, organic dyes, drugs, etc.

The electrochemical properties were investigated by recording polarization curves using a potentiostat P-5848 (NTUU “KPI”, Kyiv, Ukraine) in potentiodynamic mode with a scan rate of 0.2 V/s. The measured electrode potentials were converted to the hydrogen electrode potential scale.

## 3. Results

### 3.1. X-ray Structural and X-ray Spectral Analysis Results

The obtained coatings were identified based on their phase composition, chemical composition, and microhardness characteristics. The phase compositions, thickness and microhardness of the chromium–aluminum coatings on the surface of Ti-6Al-4V are summarized in Table 1.

**Figure 1:**
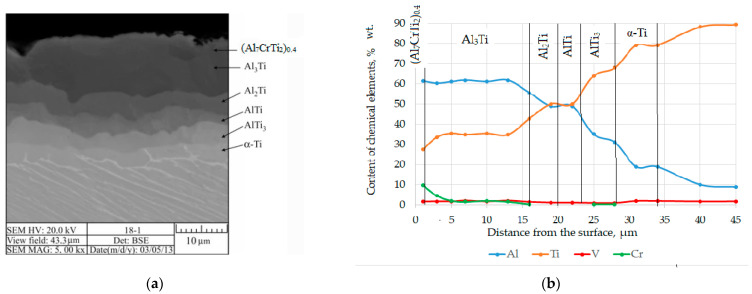
Microstructure (**a**) and distribution of elements (**b**) on the surface of titanium alloy Ti-6Al-4V after chromaluminizing (temperature—1050 °C; time—4 h).

**Figure 2:**
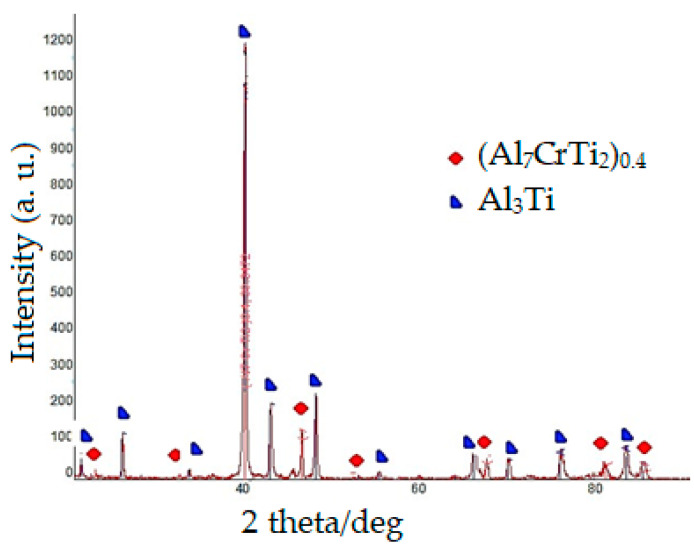
X-ray diffractogram of the surface of titanium alloy Ti-6Al-4V after chromaluminizing (temperature—1050 °C; time—4 h); Cu_Kα1_ radiation, wavelength—0.154 nm.

X-ray structural analysis established that on the surface of the Ti-6Al-4V titanium alloy after chromaluminizing (Table 1 and Figure 1a), the presence of peaks corresponding to phases based on Al_3_Ti, (Al_7_CrTi_2_)_0.4_ is recorded. According to the micro-X-ray spectral analysis data (Figure 1b), it was established that the surface layer of the coating contains 61.3–60.24% wt. aluminum, 27.5–33.62% wt. of titanium, 1.5–1.64% wt. vanadium, and 9.7–4.5% wt. chromium. The obtained data correlate well with X-ray structural analysis (Figure 2) data, and this layer was identified as an Al_3_Ti-based phase slightly doped with chromium.

It should be noted that the maximum amount of chromium (9.7–4.5% wt.) is observed precisely in the surface layer of the coating. As the coating progresses deeper, the amount of chromium decreases, and at a distance of 15 microns, its content does not exceed 1.5% wt.

The content of aluminum in the surface layer based on Al_3_T remains almost unchanged and amounts to 61.3–60.24% wt., which is due to the insignificant area of homogeneity of this compound [39]. As the coating progresses, the amount of aluminum gradually decreases and amounts to 55.5–19.1% wt.

It should be noted that the presence of chromium in the surface layer of the coating is in the amount of 9.7–4.5% wt. should help reduce the fragility of the coating [40,41].

It was established that the phases of the diffusion coating are formed in the sequence corresponding as follows: Al_2_Ti, AlTi, AlTi_3_. A zone based on α-Ti was found directly under the coating. The formation of this zone is probably associated with the diffusion of Al to the boundary of the coating-substrate. The aluminum content in this zone is 20.1–19.1% wt.

Microstructural analysis established that the resulting coatings consist of continuous layers separated by a straightforward interface. The surface phase (Al_7_CrTi_2_)_0.4_, recorded by XRD analysis, is not microstructurally separated from the Al_3_Ti phase. No chips, cracks, or delaminations were detected along the coating cross-section (Figure 1a). The obtained data on the phase and chemical composition of the coatings correlate well with the study [15], which was carried out by the authors of this work the day before.

The layer-by-layer X-ray phase analysis of the surface of titanium alloy Ti-6Al-4V after chromaluminizing (Table 1 and Figure 2) revealed the phase (Al_7_CrTi_2_)_0.4_. After that, a diffusion coating is formed, consisting of titanium aluminides: the outer layer is based on Al_3_Ti compound, the intermediate Al_2_Ti layer is located under the AlTi layer, and the AlTi_3_ titanium aluminide is directly adjacent to the base. In this case, the phases of the diffusion layer are formed in the sequence determined by the isothermal section of the Al-Ti diagram of state at the temperature of chemical-heat treatment [42].

Thus, the chromoaluminizing changes the chemical composition, phase composition, and structure of the surface layers of the Ti-6Al-4V alloy. A diffusion coating with a total thickness of 32–34 µm is formed on the alloy surface. The phase composition gradually changes from the (Al_7_CrTi_2_)_0.4_ phase to Al_3_Ti, Al_2_Ti, AlTi, AlTi_3_, and a solid solution of the α-Ti phase. The microhardness of the obtained coatings is relatively high and reaches 8.0 GPa. Such a high microhardness of intermetallic layers with the participation of aluminum, titanium, and chromium is probably due to dispersed inclusions of oxides and nitrides.

### 3.2. Massometric Tests Results

The results of the massometric tests are based on the data from visual observations of the surface condition of the samples and changes in solution color after corrosion studies. Thus, in solutions of weakly dissociated acetic acid (*K_m_* = 1.76∙10^−5^ g/(m^2^∙h)) and adipic acid (*K_m_* = 3.7∙10^−5^ g/(m^2^∙h)), changes in the mass of coated and initial Ti-6Al-4 V alloy samples are insignificant.

There are no noticeable changes in the solutions’ color or the coated samples’ surface condition after corrosion testing. However, the surfaces of the uncoated samples exposed to the acetic acid solution were darkened, and on the surfaces of samples aged in adipic acid solutions, the areas of local corrosion were observed. Somewhat more considerable changes in the mass of the samples are observed in a more concentrated 10% oxalic organic acid (*K_m_* = 3.0∙10^−3^ g/(m^2^∙h)). There is also a change in the appearance of the samples and the color of the solution: the most significant changes in the surface condition of the samples and the color of the solutions occur in the most aggressive environments, such as sulfuric acid and phosphoric acid solutions.

The conducted corrosion tests indicate that the corrosion resistance of the initial Ti-6Al-4V alloy samples and samples with chromium–aluminum diffusion coatings significantly depends on the nature of the aggressive environments (Table 2).

The samples of initial Ti-6Al-4V alloy exhibit the highest corrosion resistance in aqueous solutions of 10% Na_2_CO_3_, 15% CH_3_COOH, 40% HNO_3_, slightly lower resistance in 1.5% C_6_H_10_O_4_ and 10% C_2_H_6_O_4_, and the lowest resistance in 3% and 35% H_2_O_2_, 10% H_3_PO_4_, and 5% and 10% H_2_SO_4_.

A selective effect of an aggressive environment on the corrosion resistance of the Ti-6Al-4V titanium alloy [43] corresponds to the role of various factors:

- the protective properties of oxide films that can form on the surface of the Ti-6Al-4 V titanium alloy may selectively affect its corrosion resistance in solutions of reducing acids (such as H_3_PO_4_ and H_2_SO_4_) due to chemical dissolution during the interaction of H^+^ ions, the protective properties of such films can significantly decrease, in solutions containing oxygen or other oxidizing agents (e.g., H_2_O_2_, HNO_3_) increase;
- different types of depolarization of corrosion processes are as follows: oxygen depolarization in Na_2_CO_3_ solutions, oxygen or hydrogen-oxygen depolarization in organic acid solutions, hydrogen depolarization in H_3_PO_4_ and H_2_SO_4_ solutions, oxidizing in H_2_O_2_ solutions, hydrogen-oxidizing in HNO_3_ solutions;
- by the selective effect of anions of the environment on the rate of the anodic reaction of dissolution of metals, since anions SO_4_^2−^, HSO_4_^−^ usually catalyze its flow, and anions NO_2_^−^, CO_3_^2−^ may lead to the passivation of metals.

Table 2 contains the corrosion rate, g/(m^2^∙h):

(2) $$K_{m}=\frac{g_{2}-g_{1}}{S\cdot \tau },$$

where *g*_1_, *g*_2_—the mass of the samples before and after corrosion, respectively, g; *S*—the surface of the sample, m^2^; *τ*—duration of the corrosion process, h.

The corrosion inhibition ratio of the coating is determined as follows:

(3) $$\gamma =\frac{K_{m}}{K_{m}^{*}},$$

where *K_m_*, K_m_^*^—corresponding massometric indicators of the corrosion rate for the initial alloy sample and with coating, respectively, g/(m^2^∙h).

Also, the protection degree is introduced:

(4) $$PD=\frac{K_{m}-K_{m}^{*}}{K_{m}}\cdot 100\%=\left(1-\frac{1}{\gamma }\right)\cdot 100\%.$$

The corrosion resistance of Ti-6Al-4V samples with chromium–aluminum coating in the studied environments occurs in the same sequence as for samples of the initial titanium alloy, except for solutions of 10% Na_2_CO_3_ and 40% HNO_3_. It gradually decreases in the following series: 10% CH_3_COOH > 1.5% C_6_H_10_O_4_ > 5% H_2_O_2_ > 10% NaCO_3_ > 10% C_2_H_2_O_4_ > 35% H_2_O_2_ > 10% HNO_3_ > 10% H_3_PO_4_ > 10% H_2_SO_4_. The effect of chromaluminizing on the corrosion resistance of samples is also different and depends on the nature of the aggressive environment.

Thus, the obtained chromium–aluminum coatings lead to an increase in the corrosion resistance of Ti-6Al-4V titanium alloy by 1.5–2.0 times in solutions of acetic and oxalic acids, up to 9.5 times in hydrogen peroxide solutions and by 13.8 times in the environment of the adipic acid. The protection degrees against corrosion of coated Ti-6Al-4V samples in these solutions are 34–50, 43–89.5, and 92.7%, respectively. However, samples with chromium–aluminum coatings have less corrosion resistance in sulfate, nitrate, and phosphate acid solutions than the initial titanium alloy samples.

### 3.3. Voltammetric Test Results

**Figure 3:**
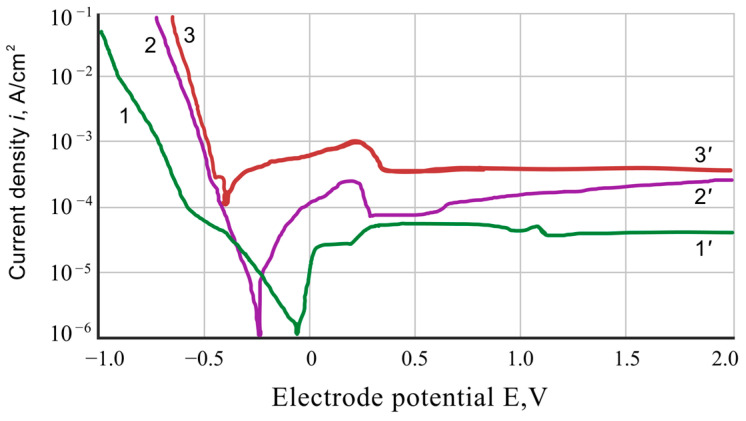
Polarization curves for 5% aqueous solutions of sulfuric acid: 1-1′—initial Ti-6Al-4V alloy; 2-2′—Ti-6Al-4V alloy with chromium–aluminum coatings; 3-3′—pure aluminum alloy.

**Figure 4:**
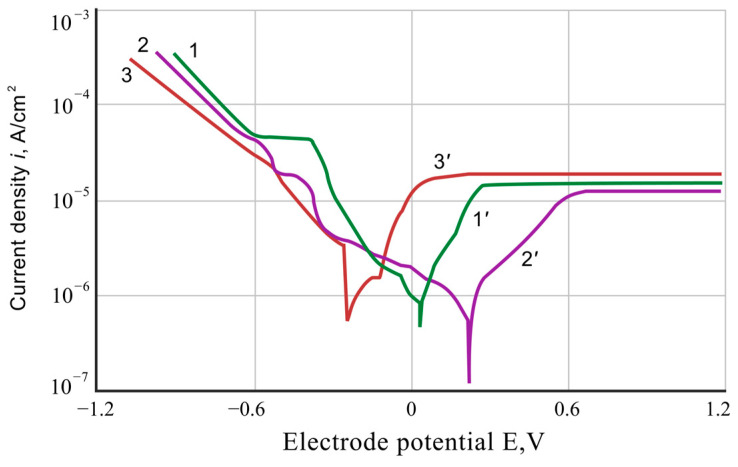
Polarization curves for 1.5% adipic acid solution: 1-1′—initial Ti-6Al-4V alloy; 2-2′—Ti-6Al-4V alloy with chromium–aluminum coatings; 3-3′—pure aluminum alloy.

**Figure 5:**
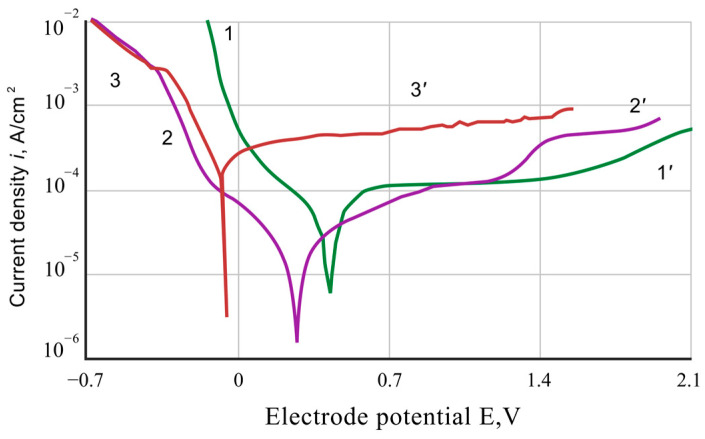
Polarization curves for a 12% hydrogen peroxide solution: 1-1′—initial Ti-6Al-4V alloy; 2-2′—Ti-6Al-4V alloy with chromium–aluminum coatings; 3-3′—pure aluminum alloy.

The results of the voltammetric investigations indicate a significant influence of the chromaluminizing treatment on the electrochemical properties of Ti-6Al-4V alloy electrodes (Figure 3, Figure 4 and Figure 5).

In the 5% H_2_SO_4_ solution, the corrosion of both coated and uncoated samples occurs with hydrogen depolarization at potentials more negative than the equilibrium potential of the hydrogen electrode (Figure 3). The reaction describes the main cathodic reaction of the process: 2 H_3_O^+^ + 2 e = H_2_ + 2 H_2_O. The hydrogen releasing on the Ti-6Al-4V alloy samples near their Es-potential describes a linear Tafel dependence, which at higher polarizations turns into a new straight-line dependence with higher values of water overvoltage.

The process of hydrogen separation on the surface of the Ti-6Al-4V alloy with a coating in a wide range of potentials is also described by a linear Tafel dependence, which in a specific range of potentials coincides with the dependence obtained on the pure aluminum alloy electrode. The application of the coatings leads to an increase in the overvoltage of hydrogen separation near the Es-potential of the electrode and a shift of the potential values in a more negative direction compared to the initial state of the Ti-6Al-4V alloy (from −0.05 V to −0.2 V).

The anodic dissolution of uncoated and coated Ti-6Al-4V electrodes near Es-potentials occurs in the active areas, but their gradual transition to the passive state is observed at higher polarization. Applying the coatings accelerates the rate of anodic dissolution of the Ti-6Al-4V alloy in the active area by approximately 10 times, increases its critical potential, and slightly enhances the passivation current. The anodic polarization curves obtained on the coated Ti-6Al-4V alloy lay between the corresponding polarization curves of the initial Ti-6Al-4V alloy and pure aluminum alloy. This indicates that the anodic dissolution of the coated samples occurs with the participation of titanium and aluminum, accelerated by aluminum’s presence in the coating.

The current corrosion parameters of the coated alloy, determined by the intersection of cathodic and anodic polarization curves, are slightly lower than those of the original alloy, indicating a slight protective effect of the coating at the first stages of the corrosion process. This is due to the inhibition of cathodic corrosion reactions of the alloy due to the presence of aluminum intermetallics in the coating, a metal with a high hydrogen overvoltage and capable of forming a protective oxide film on the coating surface [18]. However, over time, the aluminum oxide film in sulfuric acid solutions is destroyed, and due to the high rate of its anodic dissolution, the corrosion of coated samples is accelerated.

In the 1.5% aqueous solution of adipic acid, the Es-potentials of the initial Ti-6Al-4V alloy samples and the coated ones are more positive than the equilibrium potential of the hydrogen electrode (Figure 4).

The corrosion processes in this solution occur with oxygen depolarization according to the reaction: O_2_ + 4 e + 4 H^+^ = 2 H_2_O. The cathodic polarization curves of both types of samples have a complex character: they show kinks caused by the overlay of the main process of oxygen reduction with the reactions of titanium, aluminum, or chromium oxide film reduction and at high polarizations by the cathodic release of hydrogen (Figure 4). The anodic dissolution of samples near the Es-potentials of the electrodes occurs in the active areas and at higher polarization areas of their passive state.

The saturation of Ti-6Al-4V alloy with chromium and aluminum leads to an increase in the polarization of the cathodic reaction of the corrosion process near the Es-potential of the electrode, which probably is due to the presence of continuous protective films of aluminum oxides or chromium oxides on the surface of the coatings. The anodic polarization curves of the coated titanium alloy are located between the corresponding curves of the uncoated titanium alloy and pure aluminum alloy, which indicates an acceleration of the anodic corrosion reaction of coated Ti-6Al-4V alloy due to the presence of primarily aluminum in its composition. The current corrosion index of the coated alloy, calculated from the data of polarization measurements, is several times lower than the corrosion index of uncoated samples. This correlates well with corrosion test results. The Es-corrosion potential of the coated Ti-6Al-4V alloy is more positive (+0.2 V) than the potential of the initial titanium alloy (+0.05 V).

In hydrogen peroxide solutions, the corrosion of initial Ti-6Al-4V and coated alloy samples proceeds with oxidative depolarization, and the main cathodic process is the hydrogen peroxide reduction reaction: 2 H_2_O + 3 H_3_O^+^ + 2 e = 4 H_2_O. The anodic dissolution of the uncoated titanium alloy and coated alloy samples takes place in the active area, but with slight polarizations of the electrodes, their gradual transition into a passive state occurs. The application of the chromium–aluminum coatings to the surface of Ti-6Al-4V leads to an increase in the polarization of the cathodic reaction of the corrosion process, accelerates the rate of anodic dissolution of the titanium alloy, and practically does not affect its passivation current (Figure 5). The cathodic curves taken on coated samples repeat the nature of the corresponding curves taken on uncoated samples. However, they have a more negative potential than the last ones. They agree with the cathodic polarization curves taken on a pure aluminum alloy at certain cathodic polarizations.

In the active and transitional state areas, the anodic polarization curves obtained on the coating are located between the analogous curves obtained on the initial Ti-6Al-4V samples and the aluminum alloy samples. Higher polarizations corresponding to the passive state area coincide with the respective curves of the initial titanium alloy. Therefore, the conducted investigations indicate the influence of aluminum on the rate of cathodic and anodic reactions of the coated samples and the occurrence of its anodic dissolution involving aluminum. The corrosion current of the coated samples in the hydrogen peroxide solution is several times lower than the uncoated titanium samples’ corresponding corrosion current, which correlates well with the corrosion test data. The corrosion potential of the coatings is more negative (+0.3 V) than that of the initial titanium alloy (+0.5 V).

### 3.4. Microstructural Analysis Results

**Figure 6:**
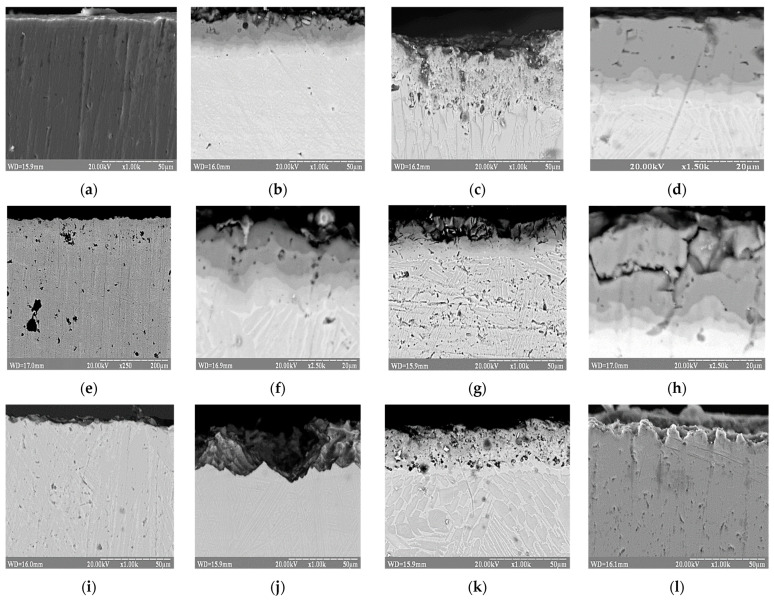
Microstructures of the uncoated (**a**,**c**,**e**,**g**,**i**,**k**) and coated Ti-6Al-4V titanium alloy samples (**b**,**d**,**f**,**h**,**j**,**l**) after corrosion: (**a**,**b**)—in 15% aqueous solution of acetic acid (CH_3_COOH); (**c**,**d**)—in 1.5% aqueous solution of adipic acid (C_6_H_10_O_4_); (**e**,**f**)—in 35% H_2_O_2_ solution; (**g**,**h**)—in 10% aqueous solution of oxalic acid (C_2_H_2_O_4_); (**i**,**j**)—in a 40% aqueous solution of nitric acid (HNO_3_); (**k**,**l**)—in 5% aqueous solution of sulfuric acid (H_2_SO_4_).

The results of microstructural studies of the surface of samples exposed to solutions of acetic and adipic acids for a long time indicate a slight uniform dissolution of coated and uncoated alloy samples, more noticeable for the last ones (Figure 6a–d).

Also, no significant damage to the layers of the protective coating was observed on the coated samples exposed to hydrogen peroxide solutions. Only isolated point damage was found in the deep layers of the alloy matrix (Figure 6e,f), which are probably related to the dissolution of vanadium as a constituent component of the Ti-6Al-4V.

Some more noticeable changes in the surface condition of the initial Ti-6Al-4V alloy and the coated samples occur in the oxalic acid solution (Figure 6g,h).

The most significant damage to the surface of coated samples is observed after their exposure to nitric and sulfuric acid solutions (Figure 6i–l). These acids intensively corrode individual areas of the coating and penetrate the deeper layers of the samples up to the matrix due to cracks in them caused by corrosion.

In addition to the type of depolarization of the corrosion process, the corrosion resistance and protective properties of chrome–aluminum coatings are also affected by the nature and solubility of aluminum corrosion products, which significantly depend on the pH of the solution. Thus, an analysis of the Pourbaix E-pH diagrams [41] built for Al-H_2_O systems shows that in the pH < 3 range, which corresponds to the corrosion of the alloy in solutions of mineral acids (i.e., H_2_SO_4_, H_3_PO_4_, and HNO_3_), the anodic dissolution of aluminum proceeds with the formation of hydrated Al^3+^ cations. They are unable to increase the corrosion resistance of the chromium–aluminum layers. In the pH > 9 range, which corresponds to a 10% NaCO_3_ solution, the corrosion of the coatings proceeds with the formation of easily soluble aluminates that also cannot inhibit corrosion. However, in the pH 3–9 range, which corresponds to corrosion in acetic or adipic acids and hydrogen peroxide solutions, the process of aluminum corrosion is accompanied by the formation of its poorly soluble hydroxide Al(OH)_3_, which can block the surface of the coating and effectively protect it from corrosion damage.

**Figure 7:**
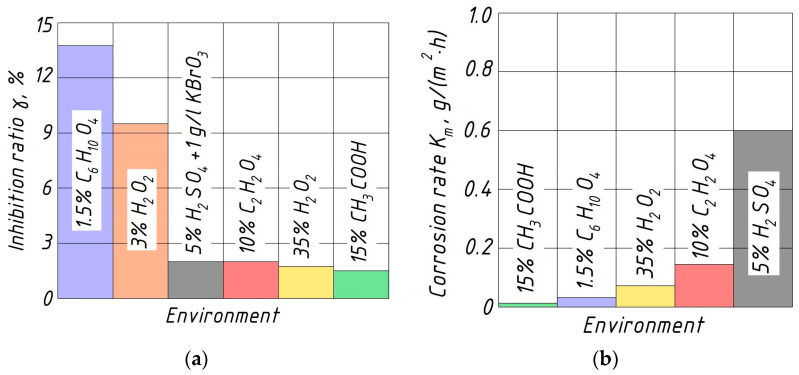
Histograms of corrosion inhibition ratio of the chromium–aluminum coatings (**a**) and corrosion rate of pure aluminum alloy (**b**) in various aggressive environments.

The above conclusions about the role of aluminum intermetallic on the electrochemical corrosion of the chromium–aluminum coatings are also confirmed by tests of the corrosion resistance of pure aluminum alloy in the studied aggressive environments (Figure 7).

The corrosion current values of the coated samples, determined by the intersection of the cathodic and anodic polarization curves, are somewhat lower than those of the uncoated samples, indicating the insignificant protective effect of the coating at the first stages of the corrosion process. This is due to the inhibition of the cathodic corrosion reactions of the titanium alloy by the presence of aluminum intermetallic in the coatings, which possesses a high hydrogen overvoltage and is capable of forming protective oxide films on the coating surface [44]. However, over time, the aluminum oxide film in sulfuric acid solutions is destroyed, and due to the high rate of its anodic dissolution, the corrosion of coated samples is accelerated [45].

## 4. Discussion

The chemical, phase composition, and structure of the surface layers of Ti-6Al-4V alloy change as a result of complex aluminochromium plating. Diffusion coatings with a total thickness of 32.0–34.0 µm are formed on the surface. The composition of the coating gradually changes from the phase (Al_7_CrTi_2_)_0.4_ to Al_3_Ti, Al_2_Ti, AlTi, and AlTi_3_. According to the data obtained, the phases of the diffusion layer are formed in the sequence determined by the isothermal section of the Al-Ti diagram of state at the temperature of chemical-thermal treatment [42]. It has been established that the surface layer of the coating contains 61.3–60.24% wt. aluminum, 27.5–33.62% wt. of titanium, 1.5–1.64% wt. vanadium, and 9.7–4.5% wt. chromium. The presence of chromium in the surface layer of the coating in the amount of more than 5% wt. should help to reduce the brittleness of the Al_3_Ti phase [39,40].

Thus, the investigations carried out in this work indicate the selective character of the corrosion resistance of uncoated and coated Ti-6Al-4V titanium alloy samples in the studied environments. They show the different effects of coatings on the corrosion of Ti-6Al-4V alloy. It was found that the samples with coatings, as well as the initial samples of titanium alloy, show the highest corrosion resistance in solutions of weakly dissociated acids (acetic and adipic), slightly lower in solutions of oxalic acid and hydrogen peroxide, and the lowest in solutions of reducing acids (sulfuric and phosphate). However, in contrast to uncoated Ti-6Al-4V samples, the chromium–aluminum-coated samples have poor corrosion resistance in 10% sodium carbonate and 40% nitric acid solutions.

Applying the chromium–aluminum coatings to the surface of Ti-6Al-4V increases the polarization of partial cathodic reactions of corrosion processes compared to the uncoated alloy in solutions of sulfate or adipic acids and hydrogen peroxide. This is primarily due to intermetallic compounds based on aluminum in the coating. This metal has a higher hydrogen overvoltage than titanium and is prone to forming solid oxide films, the protective properties of which are enhanced in oxidizing environments. In addition, the resulting coatings have a more negative Es-potential than the Ti-6Al-4V alloy in these solutions and can provide cathodic electrochemical protection of the alloy. However, if the integrity of the protective oxide films of the coating is interrupted, as in solutions of reducing acids, aluminum can be intensively dissolved, which will lead to the rapid damage of the coating layers and accelerate its corrosion. The heterogeneous nature of the coating structure and the presence of the A1_x_Ti_y_ and α-Ti phases in its composition will lead to the formation of a galvanic couple, which causes accelerated dissolution of coated samples compared to uncoated ones in reducing acid solutions. In this regard, the resulting coatings increase the corrosion resistance of the Ti-6Al-4V alloy under oxygen depolarization corrosion conditions (weakly dissociated organic acids, hydrogen peroxide) and somewhat accelerate corrosion under hydrogen depolarization conditions (H_2_SO_4_, H_3_PO_4_).

Moreover, the obtained changes in the cathodic polarization curves on the Ti-6Al-4V alloy correspond to [46]. Various factors can also explain it: the reduction in the titanium oxide film, the change in the metal’s surface charge, the desorption of acid anions (hydrogen evolution promoters) from its surface, and the formation of titanium hydrides.

Further studies will determine the heat resistance of the obtained coatings. In addition, the analysis of changes in the structure and microhardness of these protective coatings after oxidation at elevated temperatures will be studied.

## 5. Conclusions

1. The multilayer coating is formed as a result of the chromaluminizing of the Ti-6Al-4V alloy and consists of layers (Al_7_CrTi_2_)_0.4_ to Al_3_Ti, Al_2_Ti, AlTi, AlTi_3,_ and a solid solution of α-Ti The total thickness of the protective coating is 32.0–34.0 µm, and the microhardness of the obtained coatings is 2.3 times higher than the microhardness of VT6 in the initial state. It has been established that the surface layer of the coating contains 61.3–60.24% wt. Al, 27.5–33.62% wt. of Ti, 1.5–1.64% wt. V, and 9.7–4.5% wt. Cr.
2. According to the decrease in corrosion resistance of initial Ti-6Al-4V alloy and Ti-6Al-4V alloy after chromaluminizing, aggressive environments can be arranged as follows: aqueous solution of 10% Na_2_CO_3_; 15% CH_3_COOH; 10% C_2_H_2_O_4_; 35% H_2_O_2_; 5–10% H_2_SO_4_; and 10% H_3_PO_4_.
3. A lower corrosion resistance of Ti-6Al-4V alloy with chromium–aluminum diffusion coatings was recorded in comparison with the initial Ti-6Al-4V alloy in 40% aqueous solution of HNO_3_ and 10% aqueous solution of Na_2_CO_3_.
4. The chromaluminizing of the Ti-6Al-4V alloy samples leads to a 1.5–2.0 times increase in their corrosion resistance in the investigated CH_3_COOH and C_2_H_2_O_4_ solutions, 1.8–9.5 times in H_2_O_2_ solutions, and 13.7 times in C_6_H_10_O_4_ solutions. However, it is somewhat reduced in the solutions of the investigated mineral acids.
5. Corrosion of the chromaluminizing of the Ti-6Al-4V alloy, like the initial Ti-6Al-4V alloy, occurs in the studied solutions with different types of depolarization.
6. The selectivity of the corrosion resistance of the chromaluminizing coating and the nature of its influence on the corrosion resistance of samples made of the Ti-6Al-4V alloy is due to different types of depolarization of the investigated corrosion processes, as well as different protective properties of the oxide films of the coating components and the products of its corrosion destruction (and first of all, the products of aluminum corrosion).
7. The significant hardness and high corrosion resistance of the chromaluminizing alloy in solutions of organic acids and hydrogen peroxide allow it to be recommended for practical use in conditions of simultaneous exposure to titanium products of the studied oxidizing environments and the action of mechanical loads.

## Figures and Tables

**Table 1:** Phase composition and some properties of chromium–aluminum coatings on the surface of Ti-6Al-4V.

Phase Composition	Coating Thickness, μm	Microhardness, GPa
(Al_7_CrTi_2_)_0.4_	1.0–1.5	–
Al_3_Ti	11.0–13.0	8.0
Al_2_Ti	4.0–5.0	7.8
AlTi	3.0–4.0	5.0
AlTi_3_	4.0–5.0	4.0
α-Ti	4.0–5.0	3.6

Processing—chromaluminizing; the composition of the saturating mixture—Cr (28% wt.), Al (42% wt.), Al_2_O_3_ (25% wt.), NH_4_Cl (5% wt.); temperature—1050 °C; saturation time—4 h; the microhardness of the initial Ti-6Al-4V alloy—3.4 GPa.

**Table 2:** The corrosion resistance of initial Ti-6Al-4V alloy and samples with chromium–aluminum diffusion coatings in different aggressive environments (test duration—240 h, temperature—20 °C).

Environment ^1^	*K_m_*, g/(m^2^∙h)		Inhibition Ratio *γ*	Protection Degree *PD*, %
	Ti-6Al-4V Alloy	With Cr-Al Coatings		
15% CH_3_COOH, pH4	0.0091	0.0061	1.50	34
1.5% C_6_H_10_O_4_, pH3	0.1426	0.0104	13.71	92.7
10% C_2_H_2_O_4_, pH1	0.1518	0.0759	2.00	50
10% H_2_SO_4_, pH2	0.8957	0.9460	0.94	–
40% HNO_3_, pH1	0.0901	0.2120	0.74	–
3% H_2_O_2_, pH6	0.3430	0.0360	9.50	89.5
35% H_2_O_2_, pH4	0.3002	0.1715	1.75	43
10% H_3_PO_4_, pH2	0.9759	1.0460	0.93	–
10% Na_2_CO_3_, pH9	0.0017	0.0050	0.34	–

^1^ Aqueous solution.

## Data Availability

The original contributions presented in the study are included in the article, further inquiries can be directed to the corresponding author.
